# Effects of Microstructural Changes on the Phenolic Release Capacity, Volatile Profile and Color Property of Blueberry Pomace Powders at Multi-Grinding Scales

**DOI:** 10.3390/foods15193468

**Published:** 2026-09-28

**Authors:** Yang Zhang, Houmeng Liu, Yijing Yang, Lingyi Li, Wenqiang Guan, Qiang Zhang, Zhonggao Jiao

**Affiliations:** 1 Tianjin Key Laboratory of Food Biotechnology, College of Biotechnology and Food Science, Tianjin University of Commerce, Tianjin 300314, China; zy2019@tjcu.edu.cn (Y.Z.); liuhou@stumail.tjcu.edu.cn (H.L.); yangyijing@tjcu.edu.cn (Y.Y.); lilingyi@tjcu.edu.cn (L.L.); 2 Henan Key Laboratory of Fruit and Cucurbit Biology, Zhengzhou Fruit Research Institute, Chinese Academy of Agricultural Sciences, Zhengzhou 450009, China; jiaozhonggao@caas.cn

**Keywords:** multi-grinding scales, blueberry pomace, particle microstructures, phenolic release capacity, volatile profile, color property

## Abstract

Blueberry pomace (BP) powders are rich in bioactive compounds and possesses a fruity aroma, making them promising food ingredients. Micronization can improve their microstructure, phenolic release, aroma, and color. This study applied coarse grinding and superfine grinding to produce BP powders with five distinct particle sizes at three scales: plant scale (>500 μm), tissue scale (100–500 μm) and cellular scale (10–100 μm). The results and a correlation analysis indicated that at an intermediate grinding between the tissue and cellular scale, the microstructural changes of the increasing particle porosity and decreasing particle size enhanced the phenolic release capacity. However, the phenolic contents in particles at the cellular scale decreased, attributable to thermal degradation from grinding. Furthermore, a total of 45 volatile organic compounds (VOCs) were identified. Notably, cellular grinding scale enriched both fruity and roasted aroma associated with the increasing pore volume (PV) and the specific surface area (SSA) and the decreasing particle size. In addition, grinding enhanced powder lightness and redness with the control. In conclusion, considering the microstructures, functional components, aroma and color, grinding at 20 min with the particle size mostly between 10 and 100 μm, with a small portion between 100 and 200 μm, is the preferred method for blueberry pomace.

## 1. Introduction

With the rapid development of the blueberry processing industry, blueberry pomace (BP), as a major solid by-product of juice and wine processing, is generated in increasingly large quantities. BP accounts for 20–30% of the weight of fresh blueberries and retains a large amount of the bioactive ingredients, aroma and taste of the fresh fruits [1,2,3]. However, the major applications of BP are currently subjected to low-value utilization, such as compost, feed or agricultural waste disposal, resulting in severe resource wastage [4]. BP can be dried and pulverized to produce nutrient-dense whole-food powder rich in anthocyanins, dietary fiber and intense aroma. Recent research further reveals that the intact whole-food matrix preserves structural components that facilitate gradual polyphenol release and potential colonic delivery, thereby extending bioactivity beyond the small intestine, making it a superior antioxidant fortifier for snacks, bakery, and condiments compared to rapidly absorbed pure anthocyanin extracts [5,6].

Nevertheless, the food powder produced by conventional hot-air drying and ordinary pulverization methods exhibits rough mouthfeel, weak infusion capacities, bland taste and aroma, leading to poor product quality and low consumer acceptance [4,7,8,9]. Powder production involves preliminary preparation steps (e.g., cleaning, sorting, cutting and peeling), drying methods (e.g., hot-air drying, freeze drying and microwave drying), and milling conditions (e.g., ball milling, hammer milling and jet milling) [7]. Current research on BP powders focuses on the drying pretreatment [9], the contents of antioxidant compounds and dietary fibers as well as the functional properties, including the swelling, holding and retaining capacities [7]. Recently, more and more research reveals the fact that both particle size and particle microstructures (including surface area, element and morphology, the porosity, cellulose crystallinity) play an important role in altering food powders properties, which are related to different grinding scales, i.e., plant scale > 500 μm, tissue scale 100–500 μm and cellular scale 10–100 μm [10,11,12,13]. In addition, many recent studies show that the aroma profile of food powders, such as matcha powders and safflower powders, can also be affected at different grinding scales [14,15]. Moreover, color perception is another decisive factor in consumer acceptance for powder product. The degree of particle size reduction significantly affects the product’s color. Based on our previous study of black tea powder with different particle sizes, as the particle size decreases, the specific surface area increases, generally enhancing light reflection and thereby increasing lightness (L*), while fine grinding, especially at a cellular grinding scale, can disrupt cell structures and release intracellular pigments or phenolic aggregates, further modifying color attributes [13]. Given that color perception is one of the key factors influencing the consumer acceptance, understanding how processing-induced particle size changes affect the visual properties of food powders is essential for product optimization.

Ball milling has widespread applications to produce multiscale food powders with modified microstructures, enhanced aroma and functional properties [14,16,17]. However, its specific effects on the microstructures, the phenolic release capacity and aroma profile of BP powders remained unexplored.

The objective of this study was to investigate the effects of multiscale grinding scales (plant, tissue, cellular scales) on the particle microstructure, and the consequent influences on the phenolic release properties and the aroma profile of blueberry pomace powders. Moreover, the correlation between microstructural parameters, phenolic releasing contents and the changing VOCs were established for the further analysis of the mechanism. The results are expected to make a further insight for the oriented processing of value-added BP powders.

## 2. Materials and Methods

### 2.1. Raw Materials

A total of 10 kg fresh blueberries fruits (*Vaccinium corymbosum*, cv. Bluecrop) that were free of mechanical damages were purchased from a market (Tianjin, China) in 2025. The preparation for the blueberry pomace was based on the method of our previous study [9]. The fruits were crushed and pressed using a laboratory-scale screw juicer (Hengbin Machinery Co., Ltd., Weifang, China) at a screw speed of 70 r/min. During juicing, the liquid fraction passed through 2 mm sieves fitted at the side and bottom of the extractor, while the pomace was retained in the container and collected from its bottom. After juicing, about 3 kg of wet blueberry pomace was collected and hot-air-dried at 40 °C for 2 days to obtain roughly 800 g of dry pomace with a final moisture content of approximately 7% (w.b.). For each grinding treatment, 40 g of dry pomace was sampled.

### 2.2. Preparation of Blueberry Pomace Powders at Multi-Grinding Scales

For plant-scale grinding, about 40 g of the BP were coarsely ground by an RT-34 grinder (Hongquan, Hong Kong, China) with a 1.00 mm sieve. The collected sample were labeled as CBP.

Meanwhile, for tissue and cellular grinding, 40 g of the BP were pre-frozen under −80 °C for 24 h and then mixed with zirconia beads (3–10 mm in diameter) in the volume ratio of 1:2 in a UBE-F2L omnidirectional planetary ball mill (Tianchuang Powder Technology Co., Ltd., Changsha, China) with the rotation speed at 450 rpm. The ball milling process was set as 5, 10, 20 and 30 min with the samples denoted as SBP5, SBP10, SBP20 and SBP30, respectively. The ball milling procedure lasted for five minutes with intervals of five minutes to reduce the accumulation of heat.

All the obtained BP powder samples were immediately separately sealed in light-proof plastic bags and stored at −4 °C for further experiments. In addition, the VOCs of the BPs were determined within 3 days of ball milling.

### 2.3. Microstructural Characterization

#### 2.3.1. Measurement of Particle Size Distributions

The particle size of the pulverized BP powders was determined by laser light scattering using a Mastersizer 3000 particle analyzer (Malvern Instruments Ltd., Worcestershire, UK), using deionized water as the dispersion agent. The values of *D*_10_, *D*_50_ and *D*_90_ are used to characterize the particle sizes. Furthermore, the particle span, representing the width of the particle size distribution, was calculated by the following equation:

Span = (*D*_90_ − *D*_10_)/*D*_50_ (1)

#### 2.3.2. Determination of Pore Size Distributions (PSDs) and Specific Surface Area (SSAs)

Prior to the measurements, samples of BP powder were outgassed at 90 °C for 24 h. Based on the method of Liu et al. [18], the pore size distributions (PSDs) and specific surface area (SSAs) of the samples were measured with a Micromeritics Tristar II instrument (Micromeritics, Norcross, GA, USA) using nitrogen adsorption/desorption experiments. The range of determined pores was below 200 nm. The pore size distribution, average pore size, and total pore volume were determined by applying the Barrett-Joyner-Halenda (BJH) method while the SSAs were obtained by Brunauer-Emmett- Teller (BET) method in accordance with the report of Li et al. [10].

### 2.4. Analysis of Phenolic Release Capacity

A sample of 0.5 g BP powder and 75 mL pure water (25 °C) were mixed thoroughly and the mixture was put into a water bath at 25 °C to monitor the release capacity of the main phenolic contents with minor modifications regarding the water extracting temperature [12]. The mixture was briefly stirred manually with a glass rod for 10 s to ensure adequate contact between the sample and the solvent. The water extracts were produced by filtering with 0.45 μm films after1, 3, 5, 10, 20, and 30 min of leaching for the determation of the released polyphenols and flavonoids.

The analysis of polyphenols was performed by Folin–Ciocalteu method using an Evolution 201 UV-vis spectrophotometer (Thermo Fisher Scientific, Waltham, MA, USA) with the absorbance set at 765 nm. The results were calculated as gallic acid equivalents (GAE) according to the standard curves from gallic acid standards, and were reported as g/100 g on the dry basis [9].

The flavonoids content of the samples was determined as reported [19]. After diluting 30-fold, the extract of 2 mL was mixed with 0.3 mL of Al(NO_3_)_3_ (10%, *w*/*v*) and 0.3 mL of NaNO_2_ (5%, *w*/*v*). After 6 min, 2 mL of NaOH (4%, *w*/*v*) was added. The mixture was incubated for 15 min at room temperature and then analyzed at 510 nm by a UV-vis spectrophotometer. The flavonoids results were expressed in grams of rutin equivalents (RE) per 100 g of the dried samples through calibration curves with RE.

### 2.5. Volatile Profile

The flavor and volatile profile were characterized by HS-GC-IMS (FlavourSpec^®^, G.A.S, Dortmund, Germany) equipped with a MXT-5 capillary column (15 m × 0.53 mm × 1 μm, Restek, Beijing, China). The method of the detection condition were in accordance with our previous study [9]. Briefly, BP powder samples (2 g) were weighed into a 20 mL headspace bottle. The conditions of headspace sampling were as follows: Incubation temperature: 60 °C; incubation time: 20 min; agitation speed: 500 rpm; headspace gas volume: 500 μL; syringe temperature: 85 °C; splitless injuection. The flow rate of the carrier gas (nitrogen, 99.99%) was set as: 2 mL/min for 2 min, increased linearly to 100 mL/min within 18 min and held for 10 min. The IMS detector was operated at 45 °C and 5 kV with a nitrogen flow rate of 150 mL/min. A mixture of six ketones (analytical grade), namely 2-butanone, 2-pentanone, 2-hexanone, 2-heptanone, 2-octanone, and 2-nonanone (Sinopharm Chemical Reagent Co., Ltd., Beijing, China) were used as external standards. The VOCs in BP powder samples were identified by retrieving and comparing the data against the GC retention index database (NIST 2020) and IMS migration time database (G.A.S., Dortmund, Germany).

### 2.6. Characterization of Color Property

The surface color of the samples was measured using a colorimeter CR-400 (Konica Minolta, Tokyo, Japan) by spreading 10 g of BP powder evenly in a Petri dish with a flat and compact surface. Each sample was determined in triplicate, with the surface randomly changed between readings. The results were expressed as L*, a*, and b* values. The total color difference (ΔE*) between the control (CBP powder) and superfine blueberry pomace powder samples (SBP powders) was determined using the following formula:

(2) $${\Delta E}^{*}\;=\;\sqrt{{\Delta L}^{*2}+{\Delta a}^{*2}+{\Delta b}^{*2}}$$

### 2.7. Statistical Analysis

All the analyses were performed in triplicates. The results were expressed as the mean ± standard deviation. The data were analyzed by SPSS Statistics 26.0 software using one-way analysis of variance (ANOVA). Statistical significance was set at *p* < 0.05 by Duncan’s multiple-range tests. Principal component analysis (PCA), orthogonal partial least squares discriminant analysis (OPLS-DA) and variable influence in projection (VIP) model were established by SIMCA 14.0 software. The microstructural parameters of the mean particle size (MPS), particle span, specific surface area (SSA), pore volume (PV) and average pore size (APS), the content of the phenolic compounds extracted at 30 min and the total amount of peak areas of the increasing VOCs (IVOCs) and amount of peak areas of the decreasing VOCs (DVOCs)were selected as the data used in Spearman correlation analysis, which was carried out by Origin Pro (2024b) with the *p* < 0.05.

## 3. Results and Discussions

### 3.1. Microstructural Changes

#### 3.1.1. Particle Size Distributions

The particle size distributions of BP powders at multi-grinding scales are shown in Figure 1 and Table 1. The particle size distribution curves gradually shifted to the left, indicating a decrease in particle size with the extension of ball milling time for BP powders [20]. For “CBP” and “SBP5”, high percentage, of the large size particles were observed with the *D*_90_ > 500 μm and the *D*_50_ from 100 to 300 μm, indicated that for BP particles produced by ball milling within 10 min, the majority of the particles were at plant and tissue scales. Most of the BP matrix still remained intact with some tissue became separated. For “SBP10”, the mean particle size (*D*_50_, MPS) significantly decreased to 32.23 (cellular scale) while the *D*_90_ was still > 500 μm (plant scale). The grinding BP powders at this stage exhibited significant heterogeneity with the simultaneous presence of coarse and fine particulates. This inhomogeneity was primarily due to the preferential comminution of the softer, more brittle fractions, whereas the ligneous and fibrous constituents remained largely intact owing to their greater mechanical resilience [20,21]. For “SBP20”, the particle sizes ranged from 7.93 to 166.33 μm (*D*_10_–*D*_90_) with the MPS of 23.20 μm (*D*_50_), corresponding to the tissue and cellular scales. Furthermore, for “SBP30”, the particle size reduced significantly to 7.47–88.30 μm (*D*_10_–*D*_90_) with the mean particle size of 21.60 μm (*D*_50_) and the particles all belonged to the cellular scale. The results indicated that with the ball milling time extended from 20 min to 30 min, the cell structure of BP particles were gradually ruptured. The reduced value of the particle span represented the increasing overall heterogeneity of the particle size. In this study, for “CBP” sample, the span value was the lowest because large particles took the main proportion. With the extension of ball milling time, more large particles were ground into small particles. The polarization of the particle scale led to a decrease in product uniformity with increasing span value by ball milling within 10 min. As the ball milling progressed, the increasing proportion of micron particles resulted in the enhanced uniformity of the BP powders. The results were consistent with other research using BP and other different food materials. According to a previous study [7], the mean particle size (*D*_50_) of BP powders decreased from 606 μm to 170 μm with a high-speed centrifugal grinder operated for 30 s and 2 min, respectively. Further, a previous study showed that superfine grinding could effectively reduce the particle sizes of apple pomace to 11–21 μm (*D*_50_) and improve the particle uniformity with the reduced span of 1.76–2.05 [22]. Similar results were observed in other food-based powders, such as matcha [14], buckwheat hull [23] and pea insoluble dietary fiber [10]. The results proved that ball milling could effectively reduce the BP particle size to micron scale with a more concentrated size distribution.

#### 3.1.2. Specific Surface Area (SSA), Average Pore Size (APS) and Pore Volume (PV)

As displayed in Table 2, significant changes in the specific surface area (SSA), pore volume (PV) and average pore size (APS) were observed among BP powders at different grinding scales. With the grinding of BP_S_, the SSA increased, especially for samples from “SBP20” to “SBP30”. Previous studies showed that the SSA was related to both external SSA (largely depended on the particle size) and internal SSA (mainly determined by the porosity) [10]. With the extension of ball milling time from 20 min to 30 min, the rupture of BP tissue and cell structure led to the significant increasing SSA by decreasing the particle size and exposing the internal pores. The results were in agreement with the work of some other researchers [10,24,25,26]. Furthermore, PV increased significantly from “CBP” to “SBP30”, which was related to the breakdown of the BP tissue and cell wall structure as well as the explosion of the internal particle pores [10].

Figure 2 shows the pore size distributions of BP powders at different grinding scales. The pore size distribution curves of all the samples were almost the same. Figure 2 and Table 2 showed that all the BP powder samples exhibited microporous structures as the presence of a high contribution of mesopores with the diameters of 2–10 nm. Ball milling increased the PV of BP powders as a result of the exposure of the internal pores on particle surface, and it reached the maximum when grinding time increased to 30 min. In addition, with the exposure of mesopores, the APS increased within 10 min and then gradually decreased.

### 3.2. Phenolic Release Capacity

The dynamic release profiles of polyphenols and flavonoids from BP powders at different grinding scales are presented in Figure 3A and 3B, respectively. Overall, the extracted amounts of both bioactive components showed similar trends and increased progressively with prolonged ball milling time within 20 min. However, for the samples ground for 30 min with all the particles at a cellular scale, the extracted phenolic components decreased, which might be governed by enzyme-catalyzed oxidation resulting from compartmental disruption, augmented oxygen accessibility associated with increased surface area, and thermal degradation during ball milling process [7,9].

In addition, the rapid and enhanced release capacity for SBP20 and SBP30 implies that ball milling at tissue and cellular scale could change the microstructures (particle size, specific surface area, pore size and volume) by cell disruption for BP powders, making the phenolic components in readily extractable soluble conjugate forms with weakened barrier effect caused by cell wall structures. However, for CBP, SBP5 and SBP10, the cell wall structures remained relatively intact in morphology. The phenolic components distributed within the cell wall matrix and some phenolic compounds were in binding forms, leading to the limited release capacities. Further, regarding treatment efficiency, SBP20 consistently yielded the highest extraction values for both polyphenols and flavonoids. Statistical analysis further revealed distinct patterns between the two bioactive components. For polyphenols, SBP20 and SBP30 did not differ significantly at 20 min, and SBP10 and SBP30 showed no significant difference at 30 min, indicating that prolonged milling beyond 20 min provided no statistically significant gain in phenolic release. In contrast, for flavonoids, significant differences were observed among all samples at each leaching time, suggesting that the release of flavonoids remained sensitive to grinding scale throughout the milling process. These results indicated that excessively prolonging milling beyond 20 min offered negligible improvement for polyphenols and flavonoids. Taking into account both energy consumption and extraction efficacy, grinding to a particle size intermediate between the tissue and cellular scales is identified as the most favorable method for BP processing, thereby achieving a balanced release of bioactive compounds while avoiding unnecessary energy expenditure from over-milling.

### 3.3. Analysis of Volatile Organic Compounds (VOCs) Using GC-IMS

#### 3.3.1. Qualification of VOCs in BPs

GC-IMS was applied to analyze the volatile organic compounds (VOCs) in BP powders at different grinding scales. The results indicated that a total of 45 VOCs were detected in the BP powder samples (Figure 4 and Table 3), including 12 alcohols, 8 aldehydes, 6 esters, 5 pyrazines, 4 ketones, 4 acids, 3 phenolic, 2 furans and 1 terpene.

Referring to the peak signal of the topographic map, the fingerprints of the BP powder sample were obtained (Figure 4). The result of the fingerprint analysis clearly showed that the concentration of the same volatile compounds in BP powder samples significantly influenced by grinding scales with different microstructures. With the extension of grinding time, part of the VOC level in BP powder samples gradually decreased (labeled with yellow box), while some of the compounds continually increased (labeled with red box). The results showed that the content of 3-sec-butyl-2-methoxypyrazine, α-terpineol, decanal, 1-menthol, diethyl butanedioate and 2-methylisoborneol decreased significantly for BP powders with the extension of grinding time, while the contents of sesamol, ethyl heptanoate, 2-hexanol, isopentyl acetate, 4-methylphenol, 2-ethylpyrazine, furaneol, 2-3-diethyl-5-methylpyrazine and pentanoic acid increased. Previously, several research revealed the fact that the microstructures of matcha, dietary fiber, shiitake mushrooms and safflower, including particle size, specific surface area and porosity, played an important role in the flavor formation, preservation and release capabilities [9,15,27,28]. In this study, when ball milling for 30 min at a cellular scale, the particle size distribution (*D*_10_–*D*_90_) of the BP powders decreased to 7.47–88.30 μm, indicating that the plant matrix of the pomace was broken by separating and damaging the tissues and cell structures [12]. In addition, the SSA was increased significantly with the exposure of internal mesopores and macropores. The changes in the microstructures led to an easier release of volatile metabolites, resulting in the increasing categories and contents of VOCs detected by GC-IMS for BP powders. Notably, when the pulverization continued after 20 min, it was found that the mean particle size of the powder did not change significantly, but the contents of VOCs indicated an obvious change, which might be caused by Maillard reaction, lipid oxidation, thermal decomposition due to the accumulated heat produced during ball milling process [27,28,29,30]. This evidence revealed that the type and content of volatile aroma compounds in BP powders changed during ball milling process, implied that the formation of aroma for BP powders was a dynamic process. Furthermore, according to our previous study, the VOCs of BP powders dried at high temperatures ≥60 °C could enhance the unique floral and fruity aroma of BP powders [9], which was in accordance with this study that the accumulated heat produced with the increasing grinding time (≥20 min) could help enrich the aroma of BP powders.

#### 3.3.2. Multivariate Statistical Analysis

The PCA score plot is shown in Figure 5A. PC1 and PC2 explained 44.9% and 17.9% of the total variance, respectively, with a cumulative contribution of 62.8%, indicating that the first two principal components captured the major variation in the VOC profiles of BP powders subjected to different grinding treatments. The samples subjected to prolonged ball milling showed distinct distribution patterns in the PCA score plot, suggesting changes in their overall VOC profiles. These changes might be associated with the progressive disruption of tissue integrity and cellular structure during prolonged grinding. In contrast, BP powders subjected to ball milling for up to 10 min exhibited relatively similar distributions, which may be related to the comparatively limited disruption of cellular structures at the early stage of grinding. Notably, there was a considerable and statistically significant distance between the samples ground after 20 min, suggesting that the disruption and degradation of tissue integrity, accompanied by the collapse of cellular architecture resulted in a significant difference in VOCs. In contrast, the BP powders produced by ball milling within 10 min with the separation of plant tissues and minimal cellular disruption showed close similarities.

For the identification of the characteristic VOC markers in BP powders at different grinding scales, a supervised orthogonal partial least squares discriminant analysis (OPLS-DA) was carried out. Figure 5B shows the OPLS-DA scores of the VOCs in BP powders. The examination indexes (R^2^X = 0.960, R^2^Y = 0.947 and Q^2^ = 0.727) indicated a superior forecast capacity of the model. Meanwhile, the regression line for Q^2^ (−1.2) was below zero (Figure 5C), implying a reliable and stable simulation. The results were similar with the PCA scatter diagram.

The VOCs with values of variable importance in projection (VIP) > 1 were regarded as potential discriminant marker between the samples [31]. According to Figure 5D, the VIP values of the following 20 categories VOCs were >1, including 5 alcohols (phenylethyl alcohol, citronellol, 1-octanol, 2-octanol, and 3-octanol), 3 aldehydes (heptanal, 2-phenylacetaldehyde and decanal), 3 pyrazines (2-5-dimethylpyrazine, 2-ethylpyrazine and 2-3-diethyl-5-methylpyrazine), 3 ketones (2-hexanone, 1-Octen-3-one and furaneol), 2 esters (diethyl butanedioate and isopentyl acetate), 2 acids (pentanoic acid and hexanoic acid), and 2 furans (2-acetylfuran and 2-pentylfuran). These compounds played an important role in distinguishing the BP powders produced by ball milling at different grinding scales.

### 3.4. Color Property

The color parameters (L*, a*, and b*) and total color difference (Δ*E*) of BPs at different grinding scales are presented in Table 4. The results indicated that grinding time significantly affected all three color attributes (*p* < 0.05). The L* value increased from 45.85 ± 0.90 in CBP to 52.30 ± 1.11 and 53.28 ± 0.62 in SBP5 and SBP10, respectively, before decreasing to 49.07 ± 0.39 and 48.42 ± 0.79 in SBP20 and SBP30. The a* value followed a similar pattern. In contrast, b* remained relatively stable at 1.66–1.71 from SBP5 to SBP20, but decreased to 1.38 ± 0.08 in SBP30, which was not significantly different from CBP. Using CBP as the control, the Δ*E* values of SBP5, SBP10, SBP20, and SBP30 were 9.41, 11.11, 7.35, and 7.13, respectively, all exceeding the visual threshold (Δ*E* > 3), which was generally considered to indicate a perceptible color change [32]. The maximum Δ*E* at SBP10 was consistent with the highest L* and a* values, whereas the minor change in b* indicated that the total color difference was mainly driven by increased lightness and redness.

These color changes were related to the physical and chemical effects of grinding. Moderate grinding (5–10 min) reduced the particle size, increased the specific surface area, and altered light reflection and scattering, thereby enhancing lightness. It also disrupted the cell walls, promoting the release and surface exposure of inner anthocyanins and other pigments, which intensified redness. In contrast, excessive grinding (20–30 min) might generate localized heat, resulting in the partial degradation of heat-sensitive anthocyanins, Maillard reactions and possible particle aggregation, leading to the decreases in L*, a*, and Δ*E*, which was similar with a reported for purple potato flour [33]. Overall, grinding time exerted a non-monotonic effect on the color properties of BPs, and 10 min was the optimal grinding time for improving powder lightness and redness while maximizing the total color difference.

### 3.5. Correlation Analysis

Figure 6 illustrates the correlations among microstructures, phenolic release capacity, and the decreasing and increasing VOCs in BP powders at different grinding scales. MPS was significantly negatively correlated with SSA and PV (*p* < 0.05), while PS was positively correlated with APS. These relationships reflected the progressive structural evolution of BP particles during ball milling. At the early stage, grinding mainly reduced particle size and separated plant tissues. As milling continued, cell walls and intracellular structures were disrupted, exposing internal pores and increasing SSA and PV. The simultaneous decrease in MPS and increase in SSA/PV therefore indicated that external fragmentation and internal pore exposure occurred together. The positive correlation between PS and APS suggested that, in samples with broad particle size distributions, the remaining coarse particles still contained larger pores or intercellular voids, which increased the average pore size. As grinding proceeded and the proportion of fine cellular-scale particles increased, these large pores were gradually destroyed or converted into smaller mesopores, leading to a decrease in APS after 10 min.

The release of polyphenols and flavonoids was positively correlated with PV and negatively correlated with MPS. This suggested that phenolic release was governed by two complementary effects: (i) reduction in particle size shortened the diffusion path and increased the external contact area between the solvent and the powder; and (ii) rupture of cell walls and exposure of internal pores increased the accessible internal surface and pore volume, allowing intracellular and bound phenolic compounds to be more readily extracted [12]. The weakened cell wall barrier also promoted the release of soluble conjugated phenolics. These correlations explain why SBP20, which had an intermediate grinding scale between tissue and cellular levels, consistently showed the highest extraction of polyphenols and flavonoids. Although SBP30 had a smaller MPS and higher SSA/PV, its phenolic release did not further increase and even decreased. This indicates that excessive grinding beyond 20 min introduced degradation processes, such as thermal degradation, enzyme-catalyzed oxidation and increased oxygen accessibility, which counteracted the benefits of further structural breakdown. Therefore, the correlation results supported an intermediate grinding scale rather than the finest cellular scale as optimal for phenolic release.

For VOCs, the opposite correlation patterns of DVOCs and IVOCs indicate two coexisting mechanisms during ball milling. DVOCs were significantly negatively correlated with SSA and PV and positively correlated with MPS, suggesting that compounds such as 3-sec-butyl-2-methoxypyrazine, α-terpineol, decanal, 1-menthol, diethyl butanedioate and 2-methylisoborneol were progressively lost as grinding proceeded. This loss could be attributed to volatilization, oxidation and thermal degradation when the protective tissue and cell structures were disrupted and the internal pores were exposed. In contrast, IVOCs were significantly negatively correlated with MPS and positively correlated with SSA and PV. Compounds such as sesamol, ethyl heptanoate, 2-hexanol, isopentyl acetate, 4-methylphenol, 2-ethylpyrazine, furaneol, 2,3-diethyl-5-methylpyrazine and pentanoic acid increased with grinding. This increase might result from both the release of pre-existing volatile compounds trapped in the plant matrix and the formation of new volatiles through Maillard reactions, lipid oxidation and thermal decomposition. The simultaneous loss and formation of VOCs demonstrated that the aroma of BP powders is a dynamic outcome of grinding, rather than a simple retention process.

Taken together, the correlation analysis provided a mechanistic link among grinding scale, microstructure, phenolic release and VOC profiles. It showed that particle size reduction alone was not sufficient to explain the quality of BP powders. The exposure of internal pores, the increase in SSA and PV, and the degree of cell wall disruption were equally important.

## 4. Conclusions

This study clarified how ball milling at multiscale levels regulates the microstructure, phenolic release, volatile profile, and color of blueberry pomace (BP) powders, and identified the optimal grinding endpoint. Particle size decreased progressively with milling time. Grinding increased SSA from 0.26 to 0.47 m^2^/g and PV from 4.37 × 10^−4^ to 6.87 × 10^−4^ cm^3^/g, while APS first increased and then decreased, indicating progressive cell disruption and pore exposure. Phenolic release was not maximized at the finest cellular scale. SBP20 consistently yielded the highest extraction values for both polyphenols and flavonoids; extending milling to 30 min did not significantly improve polyphenol release and even reduced the extracted phenolic components, likely due to thermal degradation and oxidation. Correlation analysis supported this conclusion: MPS was negatively correlated with SSA and PV, and phenolic release was positively correlated with PV and negatively correlated with MPS. Therefore, an intermediate grinding scale between the tissue and cellular levels provides the best balance among cell wall disruption, pore exposure, and degradation. GC-IMS identified 45 VOCs, and 20 VIP > 1 categories discriminated between grinding scales. Cellular-scale grinding enriched fruity and roasted aromas via increased PV/SSA and reduced particle size. Color changes were non-monotonic, with the largest Δ*E* at 10 min, whereas 20 min still exceeded the visual threshold. Overall, 20 min ball milling is optimal under the studied conditions, producing BP powders with most particles 10–100 μm and a small fraction 100–200 μm, balancing phenolic release, aroma, color, and energy efficiency. These findings provide a theoretical basis for the micronization of blueberry pomace. Future work should validate these results at pilot scale, evaluate sensory properties and storage stability, and extend this strategy to other fruit and vegetable pomaces.

## Figures and Tables

**Figure 1:**
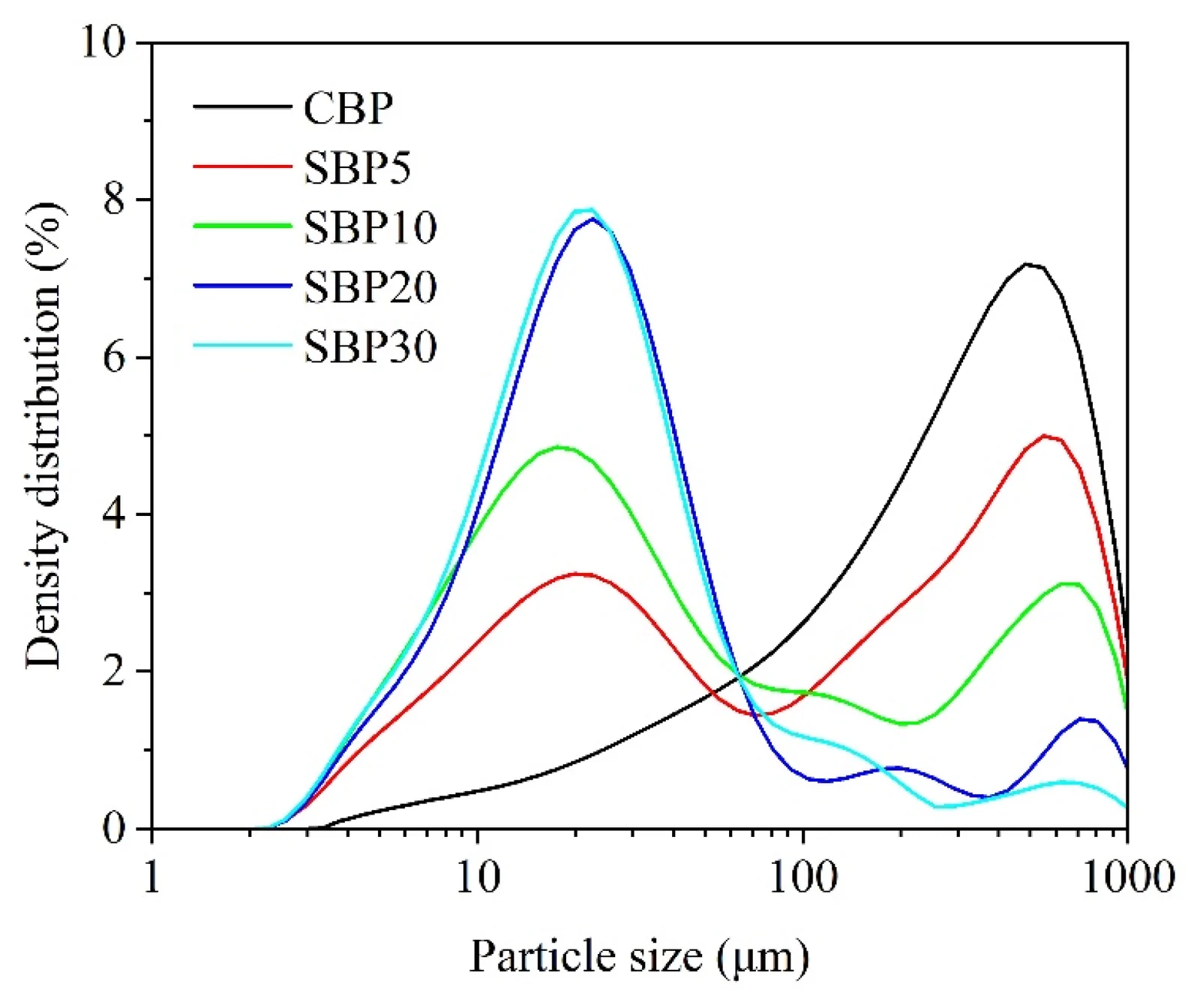
Particle size distribution curves of BP powders at multi-grinding scales.

**Figure 2:**
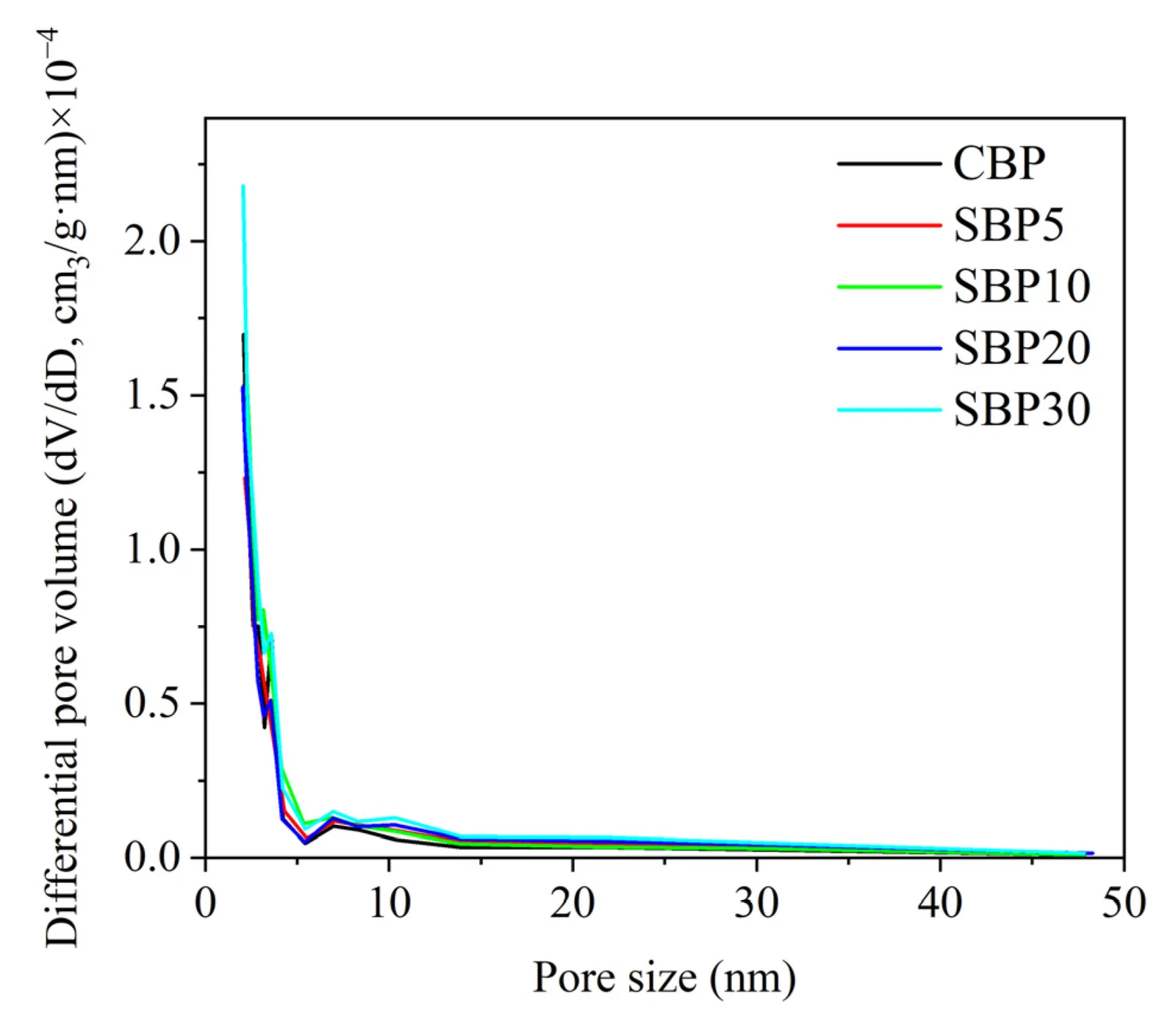
Pore size distribution curves of BP powders at multi-grinding scales.

**Figure 3:**
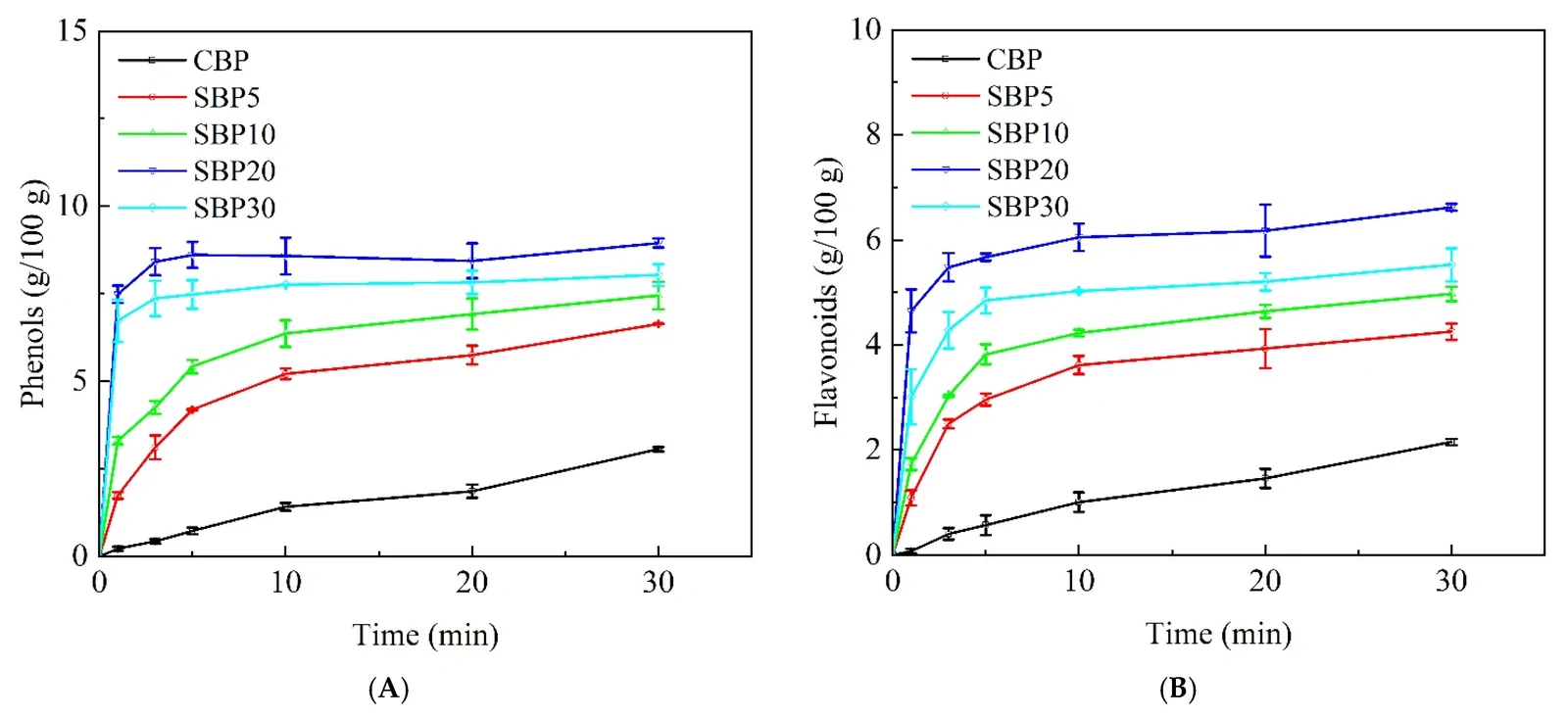
Release capacities of polyphenols (**A**) and flavonoids (**B**) of BP powders at multi-grinding scales.

**Figure 4:**
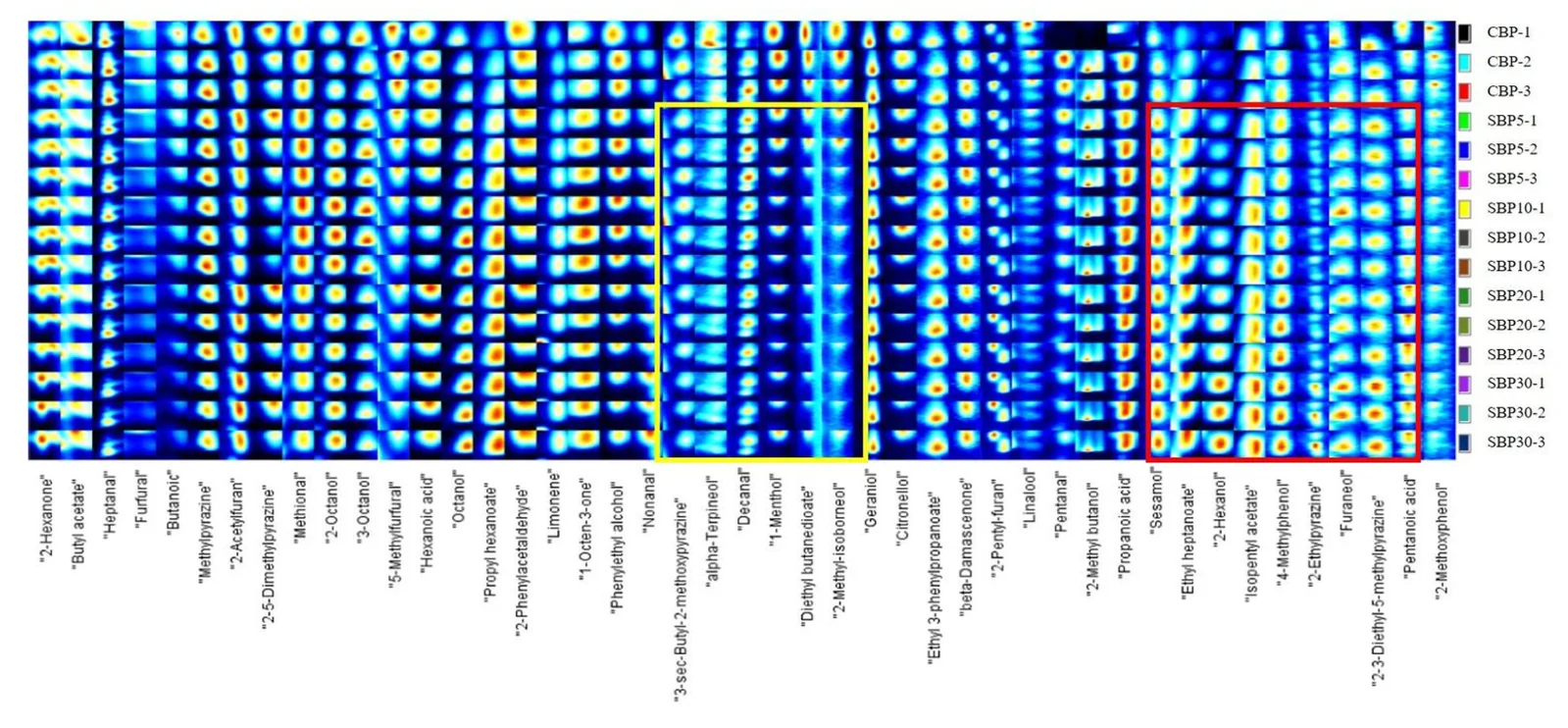
Fingerprint of VOCs of BP powders at multi-grinding scales (yellow box: VOCs decreasing with grinding time; red box: VOCs increasing).

**Figure 5:**
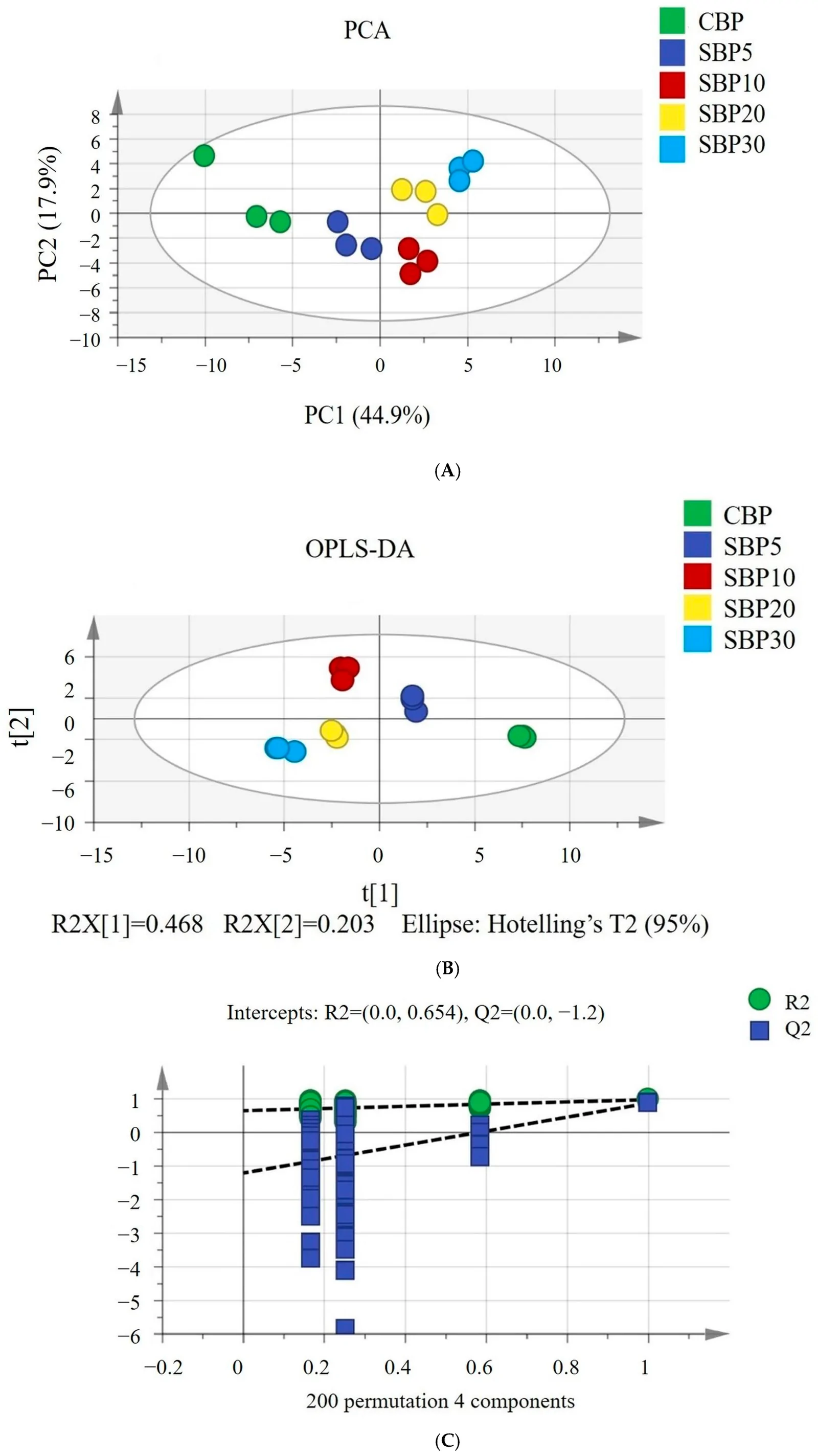

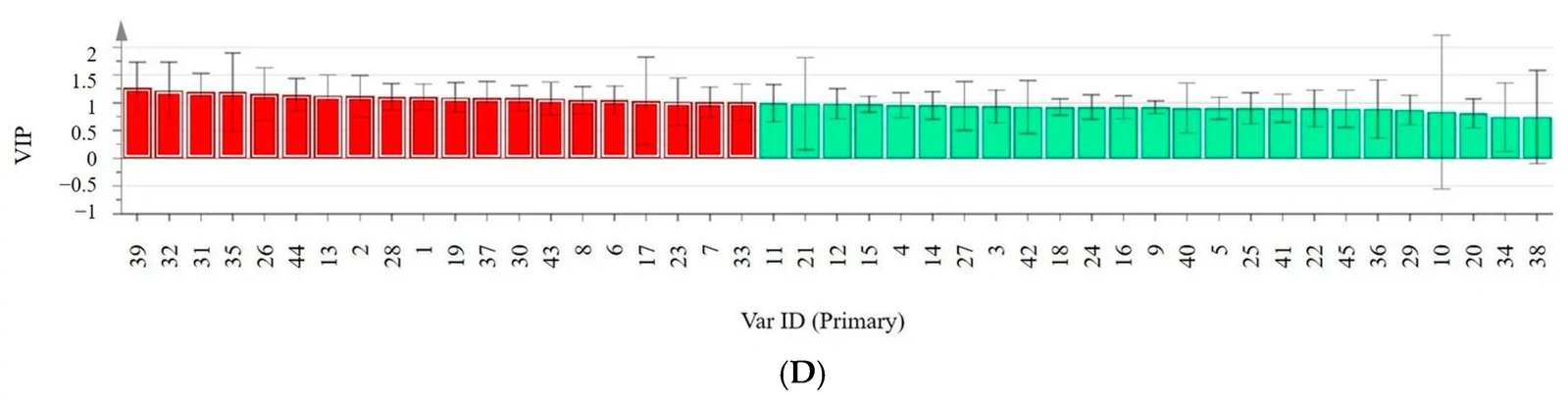
Scattering diagrams of PCA (**A**), OPLS-DA (**B**), displacement test (**C**) and VIP scores (**D**) of VOCs in BP powders at multi-grinding scales (red: VIP ≥ 1; green: VIP < 1).

**Figure 6:**
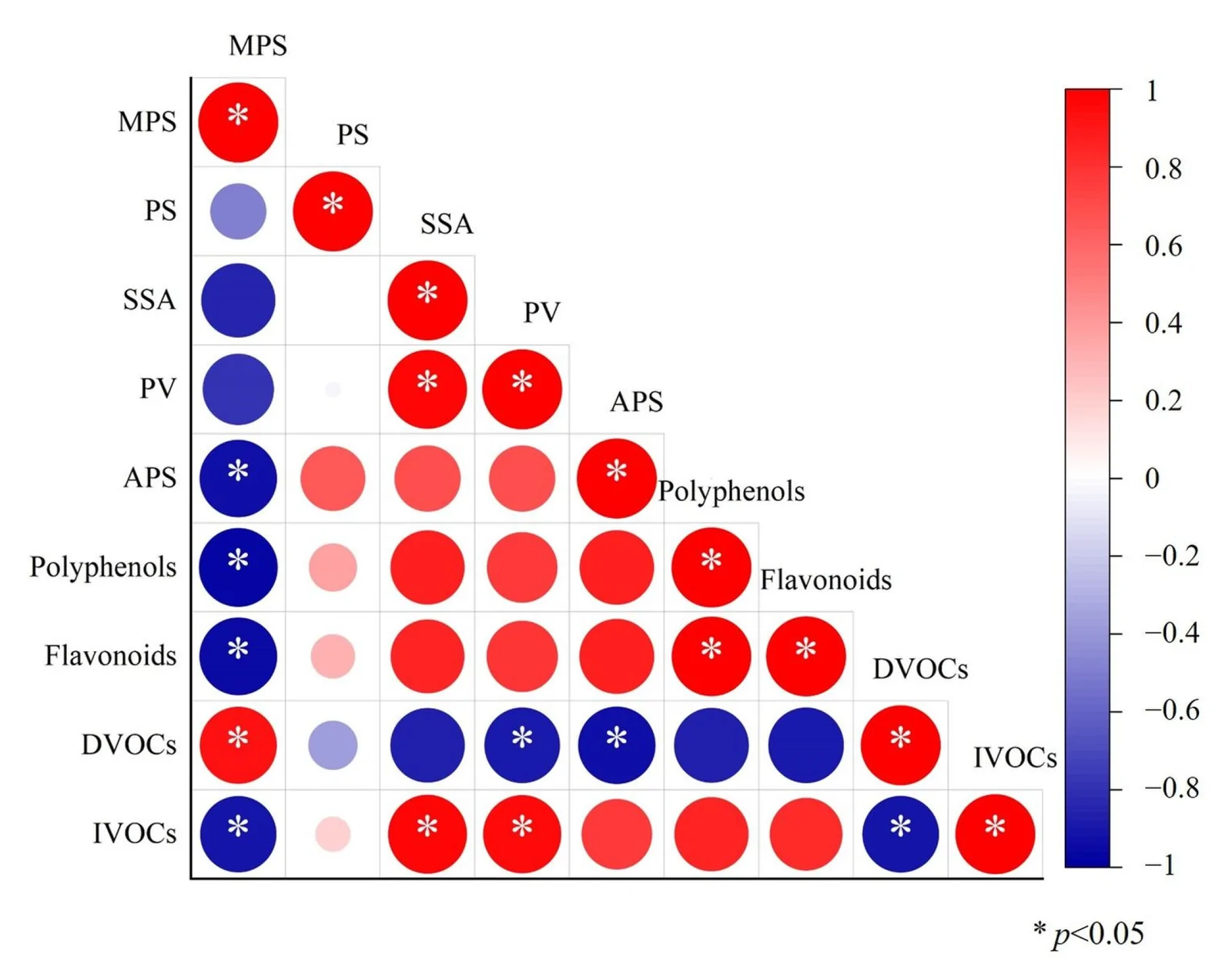
Correlation analysis of microstructure parameters with phenolic release capacity and VOCs (* indicates a significant difference at *p* < 0.05).

**Table 1:** Particle size of BPs at multi-grinding scales.

Multiscale Grinding	BP Sample	Particle Size			Particle Span
		*D*_10_ (μm)	*D*_50_ (μm)	*D*_90_ (μm)	
Plant → Tissue	CBP (0 min)	39.90 ± 1.74 ^e^	293.33 ± 6.43 ^d^	730.67 ± 10.41 ^e^	2.36 ± 0.02 ^a^
Plant → Tissue	SBP5 (5 min)	9.43 ± 0.20 ^b^	134.00 ± 8.89 ^c^	680.00 ± 17.35 ^d^	5.02 ± 0.28 ^c^
Plant → Tissue → Cellular	SBP10 (10 min)	7.50 ± 0.41 ^a^	32.23 ± 3.44 ^b^	578.00 ± 19.92 ^c^	17.87 ± 2.37 ^e^
Tissue → Cellular	SBP20 (20 min)	7.93 ± 0.19 ^a^	23.20 ± 0.66 ^a^	166.33 ± 17.10 ^b^	6.84 ± 0.91 ^d^
Cellular	SBP30 (30 min)	7.47 ± 0.25 ^a^	21.60 ± 0.52 ^a^	88.30 ± 10.85 ^a^	3.74 ± 0.46 ^b^

Values in the same column followed by different superscripts are significantly different at *p* < 0.05.

**Table 2:** The specific surface area, pore volume and pore size of BPs at multi-grinding scales.

BP Sample	SSA (m^2^/g)	PV (cm^3^/g)	APS (nm)
CBP (0 min)	0.26 ± 0.01 ^a^	(4.37 ± 0.14) × 10^−4 a^	4.86 ± 1.12 ^a^
SBP5 (5 min)	0.35 ± 0.07 ^b^	(4.88 ± 0.19) × 10^−4 b^	5.27 ± 1.47 ^ab^
SBP10 (10 min)	0.35 ± 0.08 ^b^	(5.23 ± 0.65) × 10^−4 b^	6.56 ± 0.79 ^bc^
SBP20 (20 min)	0.41 ± 0.01 ^c^	(6.00 ± 0.59) × 10^−4 c^	6.48 ± 0.77 ^c^
SBP30 (30 min)	0.47 ± 0.01 ^d^	(6.87 ± 0.10) × 10^−4 d^	6.17 ± 0.62 ^c^

SSA, specific surface area; PV, pore volume; APS, average pore size. Values in the same column followed by different superscripts are significantly different at *p* < 0.05.

**Table 3:** VOCs identified in BPs at multi-grinding scales using GC-IMS.

No.	Compounds	CAS	Formula	MW	RI	Rt	Dt	Odor Characteristics ^#^
Alcohols (13)								
1	Phenylethyl alcohol	C60128	C_8_H_10_O	122.2	1125.1	481.337	1.6529	Floral
2	Citronellol	C106229	C_10_H_20_O	156.3	1215.2	662.417	1.8191	Rose, citrus
3	α-Terpineol	C98555	C_10_H_18_O	154.3	1155.2	535.588	1.7651	Floral, herbal
4	1-Menthol	C221651	C_10_H_20_O	156.3	1177.3	579.22	1.2258	Minty
5	Linalool	C78706	C_10_H_18_O	154.3	1134.6	497.983	1.2256	Floral
6	1-Octanol	C111875	C_8_H_18_O	130.2	1077.4	406.618	1.4452	Waxy
7	2-Octanol	C123966	C_8_H_18_O	130.2	970.3	282.891	1.4542	Spicy, herbal, earthy
8	3-Octanol	C589980	C_8_H_18_O	130.2	998.3	310.168	1.3984	Earthy
9	2-Hexanol	C626937	C_6_H_14_O	102.2	971.2	283.75	1.576	Winey
10	2-Methylbutanol	C137326	C_5_H_12_O	88.1	737.6	144.826	1.2433	Winey, onion
11	2-Methylisoborneol	C2371428	C_11_H_20_O	168.3	1195.5	617.871	1.2558	Earthy
12	Geraniol	C106241	C_10_H_18_O	154.3	1243.3	731.834	1.2251	Floral
Aldehydes (8)								
13	Heptanal	C111717	C_7_H_14_O	114.2	913.4	236.136	1.3363	Green, herbal
14	Furfural	C98011	C_5_H_4_O_2_	96.1	822.6	180.909	1.3435	Bready, woody
15	Methional	C3268493	C_4_H_8_OS	104.2	945.6	261.266	1.3955	Vegetable, creamy
16	5-Methyl-furfural	C620020	C_6_H_6_O_2_	110.1	983.3	295.178	1.4924	Caramel
17	2-Phenylacetaldehyde	C122781	C_8_H_8_O	120.2	1040.1	357.288	1.5543	Green, floral
18	Nonanal	C124196	C_9_H_18_O	142.2	1116.2	466.45	1.487	Fatty, citrus, green
19	Decanal	C112312	C_10_H_20_O	156.3	1158.1	541.212	1.535	Pleasant
20	Pentanal	C110623	C_5_H_10_O	86.1	700	132.479	1.1915	Fermented, bready
Esters (6)								
21	Butyl acetate	C123864	C_6_H_12_O_2_	116.2	810.7	175.048	1.2287	Ethereal
22	Propyl hexanoate	C626777	C_9_H_18_O_2_	158.2	1080.7	411.401	1.3768	Fruity, pineapple
23	Diethyl butanedioate	C123251	C_8_H_14_O_4_	174.2	1177.9	580.508	1.2983	Fruity, apple
24	Ethyl 3-phenylpropanoate	C2021285	C_11_H_14_O_2_	178.2	1322.5	968.919	1.344	Rose, honey, fruity
25	Ethyl heptanoate	C106309	C_9_H_18_O_2_	158.2	1117.6	468.745	1.4012	Fruity, pineapple
26	Isopentyl acetate	C123922	C_7_H_14_O_2_	130.2	867	205.393	1.2862	Fruity, banana
Pyrazines (5)								
27	Methylpyrazine	C109080	C_5_H_6_N_2_	94.1	848.6	194.671	1.4131	Nutty
28	2-5-Dimethylpyrazine	C123320	C_6_H_8_N_2_	108.1	897.4	224.897	1.4875	Chocolate
29	3-sec-Butyl-2-methoxypyrazine	C24168705	C_9_H_14_N_2_O	166.2	1160	544.851	1.7101	Musty, green
30	2-Ethylpyrazine	C13925003	C_6_H_8_N_2_	108.1	931.3	249.699	1.1723	Nutty, woody, roasted
31	2-3-Diethyl-5-methylpyrazine	C18138040	C_9_H_14_N_2_	150.2	1146.4	519.091	1.2653	Musty
Ketones (4)								
32	2-Hexanone	C591786	C_6_H_12_O	100.2	803.3	171.574	1.1986	Fruity, fungal, meaty
33	1-Octen-3-one	C4312996	C_8_H_14_O	126.2	998.7	310.649	1.6931	Herbal, mushroom
34	β-Damascenone	C23726934	C_13_H_18_O	190.3	1357.6	1097.358	1.4041	Apple, rose, honey
35	Furaneol	C3658773	C_6_H_8_O_3_	128.1	1069.8	396.03	1.6038	Caramel
Acids (4)								
36	Butanoic acid	C107926	C_4_H_8_O_2_	88.1	839.2	189.51	1.3798	Cheesy
37	Hexanoic acid	C142621	C_6_H_12_O_2_	116.2	981.3	293.212	1.6335	Fatty
38	Propanoic acid	C79094	C_3_H_6_O_2_	74.1	683.1	127.492	1.0907	Pungent, acidic, cheesy
39	Pentanoic acid	C109524	C_5_H_10_O_2_	102.1	932.6	250.69	1.2225	Cheesy
Phenolics (3)								
40	Sesamol	C533313	C_7_H_6_O_3_	138.1	1317.9	953.049	1.2016	Aromatic
41	4-Methylphenol	C106445	C_7_H_8_O	108.1	1046	364.606	1.1359	Phenolic, narcissus
42	2-Methoxyphenol	C90051	C_7_H_8_O_2_	124.1	1096	434.198	1.229	Smoke, vanilla, woody
Furans (2)								
43	2-Acetylfuran	C1192627	C_6_H_6_O_2_	110.1	897.8	225.142	1.4239	Almond, caramel
44	2-Pentylfuran	C377769	C_9_H_14_O	138.2	973.9	286.238	1.2528	Fruity, green, earthy
Terpene (1)								
45	Limonene	C138863	C_10_H_16_	136.2	1003.6	315.732	1.6514	Pine, lemon

MW, molecular mass; RI, retention index; Rt, retention time; Dt, relative migration time. ^#^ Odor characteristics were obtained from the websites: https://www.thegoodscentscompany.com (accessed on 20 May 2026).

**Table 4:** Color property of BPs at multi-grinding scales.

BP Sample	L*	a*	b*	Δ*E*
CBP (0 min)	45.85 ± 0.90 ^a^	5.01 ± 0.25 ^a^	1.23 ± 0.07 ^a^	0
SBP5 (5 min)	52.30 ± 1.11 ^c^	11.84 ± 0.23 ^b^	1.66 ± 0.07 ^b^	9.41
SBP10 (10 min)	53.28 ± 0.62 ^c^	13.21 ± 0.13 ^c^	1.71 ± 0.10 ^b^	11.11
SBP20 (20 min)	49.07 ± 0.39 ^b^	11.60 ± 0.42 ^b^	1.67 ± 0.09 ^b^	7.35
SBP30 (30 min)	48.42 ± 0.79 ^b^	11.66 ± 0.38 ^b^	1.38 ± 0.08 ^a^	7.13

Values in the same column followed by different superscripts are significantly different at *p* < 0.05.

## Data Availability

The original contributions presented in this study are included in the article. Further inquiries can be directed to the corresponding authors.
